# Effect of Apple Consumption on Postprandial Blood Glucose Levels in Normal Glucose Tolerance People versus Those with Impaired Glucose Tolerance

**DOI:** 10.3390/foods11121803

**Published:** 2022-06-19

**Authors:** Yutaka Inoue, Lianne Cormanes, Kana Yoshimura, Aiko Sano, Yumiko Hori, Ryuichiro Suzuki, Ikuo Kanamoto

**Affiliations:** 1 Faculty of Pharmacy and Pharmaceutical Sciences, Josai University, Saitama 3500295, Japan; yy16313@josai.ac.jp (K.Y.); gkd2101@josai.ac.jp (A.S.); holly@josai.ac.jp (Y.H.); ryu_suzu@josai.ac.jp (R.S.); i.kanamoto@gmail.com (I.K.); 2 Department of Nutrition and Dietetics, School of Health Care Professions, University of San Carlos, Cebu City 6000, Philippines; lmcormanes@usc.edu.ph

**Keywords:** apple, impaired glucose tolerance, intake timing, postprandial blood glucose

## Abstract

The present study investigated the effect of apple consumption on postprandial blood glucose and insulin levels in subjects with normal versus impaired glucose tolerance. The study participants were ten healthy subjects with no glucose intolerance (normal subjects) (mean, 24.4 ± 4.8 years) and nine subjects with impaired glucose tolerance (mean, 45.2 ± 11.1 years, including 2 on insulin therapy). The test meal included white rice (148 g) and a Fuji apple (150 g). The normal subjects were randomly divided into two groups: the apple-first group, wherein the subjects consumed white rice 5 min after consuming the apple, and the rice-first group, wherein the subjects consumed an apple 5 min after consuming the white rice. Blood samples were then taken from both groups for 3 h. In addition, the subjects with impaired glucose tolerance received the same treatment as the normal subjects, with the difference being glucose level monitoring according to the order in which the apples were consumed. In the normal subjects, the Cmax of Δblood glucose and Δinsulin levels were 54.0 ± 5.0 mg/dL and 61.9 ± 7.2 µU/dL versus 46.2 ± 5.9 mg/dL and 49.8 ± 8.5 µU/dL in the rice-first and apple-first groups, respectively. The incremental area under the curve (iAUC) of insulin tended to decrease in the apple-first group. In the impaired glucose tolerance subjects, the Cmax of Δblood glucose was 75.2 ± 7.2 mg/dL in the apple-first group compared to 90.0 ± 10.0 mg/dL in the rice-first group, which was a significant difference (*p* < 0.05). The iAUC of blood glucose was lower in the apple-first group. Eating an apple before a meal may be a simple and effective strategy for managing the glycaemic response in individuals with impaired glucose tolerance.

## 1. Introduction

In recent years, the number of patients with type II diabetes mellitus (T2D), a lifestyle-related disease, has been increasing in Japan due to the westernization of dietary habits. Diabetes causes various complications and increases one’s risk of developing cerebrovascular and cardiovascular diseases, which are the leading causes of death in Japan [1,2]. Blood glucose levels normally rise temporarily after a meal, but continuous high blood glucose levels after a meal predispose people to diabetes, atherosclerosis, and weight gain. Although meals cause an increase in blood glucose levels, in healthy people, insulin is secreted immediately and the glucose in the blood is taken up by various organs and cells; thus, the levels return to normal within about 2 h after the meal [3]. However, non-healthy cases are difficult to control, and it is also difficult to predict blood glucose levels if insulin secretion is poor and not merely poor insulin secretion due to diet. However, poorly secreted insulin makes blood glucose levels difficult to control. High blood glucose levels after meals are associated with a higher risk of developing diabetes. Diabetes mellitus can lead to complications such as retinopathy, nephropathy, and neuropathy, the late stages of which can lead to blindness, the need for dialysis therapy, and reduced quality of life [4,5,6]. Postprandial hyperglycemia also increases the risk of atherosclerosis as it causes the production of reactive oxygen species and exposes the blood vessels to oxidative stress. The priority in the prevention of diabetes mellitus is to improve overall lifestyle, particularly exercise therapy and dietary intake. To prevent these problems, it is important to take preventive and progressive countermeasures for patients with diabetes by making dietary improvements.

The concept of the glycemic index (GI), a measure of the increase in blood glucose that occurs after a meal, has been studied extensively since it was first proposed by Jenkins et al. [7]. The authors reported that the consumption of low GI foods, which slowly increase blood glucose levels after a meal, is useful for controlling blood glucose and improving blood lipids in diabetic patients [8,9]. Controlling postprandial hyperglycemia is an important point in the treatment of T2D [10,11]. T2D mellitus is treated with a combination of diet, exercise, and drug therapy. Drug therapy using insulin secretagogues, biguanides, insulin resistance ameliorators, and alpha-glucosidase inhibitors is especially effective. Of these, alpha-glucosidase inhibitors are taken just before a meal, as evidenced by past studies, and are particularly useful for reducing postprandial hyperglycemia. They modestly decrease glycosylated hemoglobin levels and also reduce the postprandial insulin concentration of blood glucose, as evidenced by data from multiple sample timings after intake [12]. It is already known that taking other macronutrients before carbohydrates (‘preloads’, e.g., whey protein) and changing the order of intake can affect blood glucose levels [13,14]. However, it is necessary to indicate which types of foodstuffs are easily consumed by consumers in their daily lives and contribute to the control of blood glucose elevation.

Although it might be assumed that low GI foods with alpha-glucosidase inhibitory properties would be most effective when taken at the onset of a meal, little is known about them. Apples are a familiar fruit, available at low prices year-round, and highly valued for their richness in dietary fiber, vitamin C, potassium, and other nutrients. An apple is rich in organic acids, such as citric acid, and dietary fiber, which slows down the rate of gastric emptying [15,16,17], and thus might suppress the postprandial blood glucose elevation. Apples can also be cut in half and eaten easily, making them ideal for those with busy mornings and otherwise inadequate nutritional intake. As the saying goes, “an apple a day keeps the doctor away”. Apples are beneficial to the body and high in pectin, a soluble dietary fiber. Pectin is not digested or absorbed by human digestive enzymes, but it inhibits the absorption of cholesterol [18] and moderates the absorption of glucose. Thus, apples may help maintain good health [19].

We previously reported that changes in postprandial blood glucose levels depend on the order of consumption of vegetable juice and rice. The postprandial blood glucose elevation was significantly lower when vegetable juice was consumed before versus after rice [20]. We previously reported on the effect of gold kiwifruit intake timing on blood glucose levels [21]. This fruit contains glucose and fructose as carbohydrates. Unlike glucose, fructose is thought to be less likely to raise blood glucose. It would, therefore, be interesting to investigate the main carbohydrates in apples. Therefore, the consumption of apples at the beginning of a meal may effectively reduce the increase in postprandial glucose levels due to the fiber and polyphenols, which are abundant in apples, and the carbohydrate composition, fructose. It contains apple polyphenols, such as procyanidin and catechins, which have been reported to have antioxidant effects and regulate lipid metabolism. An apple’s dietary fiber includes pectin and cellulose, which may be related to the prevention of obesity and diabetes [22]. In other words, if evidence can be presented to show the effect of apple intake in healthy people and people with impaired glucose tolerance, it may contribute to dietary and nutritional guidance for the treatment of lifestyle-related diseases. It has been observed that individuals who consume antioxidant-rich foods have a lower risk of T2D [23].

The present study investigated the effects of apple consumption before versus after white rice consumption on the postprandial blood glucose and insulin profiles of subjects with normal versus impaired glucose tolerance to test its ability to contribute to the prevention of lifestyle-related diseases such as diabetes [24].

## 2. Materials and Methods

### 2.1. Subjects

Ten normal subjects without impaired glucose tolerance (four males and six females) (mean, 24.4 ± 4.8 years) and nine subjects with impaired glucose tolerance (six males and three females) (mean, 45.2 ± 11.1 years, including two on insulin therapy) were tested. The other seven patients with impaired glucose tolerance were not receiving medication to improve their blood glucose levels. This study was approved by the Josai University Ethics Review Board for Medical and Health Research Involving Human Subjects (approval number: H22-6) and conducted following the principles of the Declaration of Helsinki. The study’s purpose, content, and safety were fully explained to all subjects, each of whom provided informed consent. 

### 2.2. Test Meals

For each test, packaged white rice (148 g; Sato no Gohan; Sato Food Industry Co., Ltd., Nigata, Japan) from the same lot, prepared with 50 g of sugar, and 150 g of apple (Fuji, Japan), grated with the skin and prepared as 80 kcal for one unit, were used. The rice used in this study was a commercially cooked retort. It was only cooked in a microwave oven before meals. As it is a commercial product, the ingredients it contains are standardized by the manufacturer. The composition and energy of the nutritional components of each food are shown in Table 1. Note that the white rice (Sato no Gohan) is a retorted product and was heated in a microwave before the meal.

### 2.3. Test Protocol, Blood Sample Collection, and Analysis of Glucose and Insulin Levels

Two trials were performed on the same subjects, one with apple consumption after the intake of white rice (the rice-first group) and the other with apple consumption before the intake of white rice (the apple-first group). The study was designed as a randomized crossover experiment, with each participant taking part in both treatment orders. The test was not conducted when the female subjects were experiencing their menstrual periods. The subjects were forbidden to eat any food other than water from 21:00 the previous day until the morning of the test. The time at which they started to consume white rice (or apples) was set at 0 min; 5 min later, they consumed apples (or white rice). Blood samples were taken from the 200 µL of capillary blood sample of the fingertip using a puncture device (medisafe^®^ finetouch, MS-GN02, TERUMO, Tokyo, Japan) for self-exsanguination at 10 min before the meal and then 15, 30, 45, 31 60, 90, 120, and 180 min after the first meal. Of those samples collected, 40 µL was used for the insulin assay. Blood was collected into capillary tubes (Hematlon-L^®^: length 110 mm, diameter 2 mm, heparinized, Minato Medical Co., Osaka, Japan) and blood plasma was obtained through centrifugation.

A 40 µL sample of the supernatant solution was used to measure the plasma insulin concentration (hereafter, insulin level). For the measurement of insulin levels, 40 µL of blood was centrifuged (2610× *g*, 4 °C, 5 min) and then 25 µL of the supernatant (plasma) was immediately frozen at −80 °C. The blood glucose level was measured by the enzymatic method, while the insulin level was measured by the enzyme-linked immunosorbent assay method. Blood samples were taken 5–10 min before the meal and the values were determined at 0 min after the start of the meal (0 min values). Blood glucose levels were measured using a self-administered blood glucose measuring device (Glutest Neo Super^®^; Sanwa Kagaku Kenkyusho Co. Ltd., Aichi, Japan). Plasma insulin values were measured on 25 μL plasma samples using an insulin measuring kit (YK060 Insulin ELISA kit^®^; Yanaihara Institute Inc., Shizuoka, Japan) according to the manufacturer’s protocol. In this study, however, we did not measure the insulin levels of people with impaired glucose tolerance, as the amount of plasma collected was insufficient.

### 2.4. Statistical Analysis

The values of blood glucose and insulin over time after the intake of the test meal minus the 0 min value were labeled Δblood glucose and Δinsulin, respectively. The incremental area under the curve (iAUC) was calculated by the trapezoidal formula and used as a dynamic parameter of the blood glucose and insulin levels. The statistical analysis was performed using SPSS software (IBM, Chicago, IL, USA). The time-course of the postprandial blood glucose and insulin levels and the differences in each kinetic parameter between white rice and apple consumption was statistically analyzed using the paired t-test, with values of *p* < 0.05 considered statistically significant.

### 2.5. Determination of Carbohydrates

The carbohydrate content of the apples used in the tests was evaluated for glucose, fructose, and sucrose. The standard samples were prepared by weighing about 20 mg each of glucose, fructose, and sucrose and using distilled water to prepare 100 µg/mL samples. Subsequently, different solution concentrations (6.25 µg/mL, 12.5 µg/mL, 25 µg/mL, 50 µg/mL, and 100 µg/mL) were prepared and quantified by liquid chromatography (LC).

### 2.6. Conditions for LC Measurements

Sample measurements were performed using an electrochemical detector (ECD: SU-300, DKK-TOA) with a gold electrode, working electrode, and 1 V electric potential; 0.1 mol/L NaOH as the mobile phase; a column temperature of 40 °C; a flow rate of 0.5 mL/min; an AS8020 autosampler (Tosoh); and a sample injection volume of 10 μL. The column was an ion-exchange column (S-30/70 = St (styrene)/DVB (divinylbenzene) − 5TMDAH (tetramethyldiaminohexane), φ 4.6 mm × 150 mm, with an amine reaction on a core-shell-type filler. The limit of detection (LOD) and limit of quantification (LOQ) were calculated using the following equations.

LOD = 3.3 × (s/a)(1)

LOQ = 10 × (s/a)(2)

where s is the SD of the intercept for calibration curve and a is the slope of the calibration curve.

### 2.7. Sample Preparation for Analysis of Total Phenolics

The apple samples were cut into cubes and then freeze-dried. The samples were then lyophilized and powdered using an electric blender. The lyophilized apple sample (1 g) was extracted with 20 mL of boiled water for 1 h and the extract was filtered. The final volume of the elute was 20 mL with water.

### 2.8. Preparation of Folin Reagent and Total Phenolics

Folin’s reagent was prepared by mixing Na_2_WO_4_ (2.5 g), dodeca-molybdo-phosphoric acid (0.5 g), phosphoric acid (85%; 1.25 mL), and water (1.8 mL) and permitting the mixture to reflux for 2 h. The resulting solution was then diluted to 100 mL. The total phenolics content of dried apple was measured by a slightly modified Folin-Denis method [25]. The sample solution (50 µL) was mixed with the Folin’s regent. After 5 min, 50 µL of 10% (*w*/*v*) aqueous Na_2_CO_3_ was added, and the mixture was incubated for 2 h at room temperature. The absorbance of the reactant was measured at 700 nm using a microplate reader. Total phenolic content is shown as milligrams of tannic acid equivalent per gram of dry weight sample. The experiments were conducted in triplicate.

## 3. Results

### 3.1. Changes in Blood Glucose and Insulin Levels in Normal Subjects

**Figure 1:**
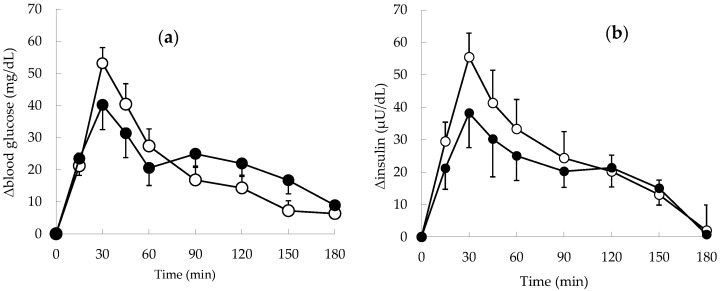
Changes in the postprandial blood glucose and insulin levels in healthy normal subjects. (**a**) Blood glucose. (**b**) Insulin. ○: rice-first group, ●: apple-first group (respectively, *n* = 10). No significant difference was noted between the rice-first and apple-first groups.

The postprandial blood glucose and insulin levels of the normal subjects in the rice-first versus apple-first groups are shown in Figure 1. The value on the vertical axis of Figure 1 represents a delta (variation from baseline). Postprandial blood glucose levels peaked at 30 min after the meal intake in both groups (Figure 1a). The 30-min postprandial value was 53.2 ± 4.9 mg/dL in the rice-first group versus 40.1 ± 7.7 mg/dL in the apple-first group, indicating the tendency of the latter to suppress the peak blood glucose level (*p* = 0.087). The rice-first group showed a rapid decrease in blood glucose levels from the peak at 30 min after the meal, while the apple-first group showed a gradual decrease.

Insulin levels followed a similar pattern (Figure 1b). In both groups, insulin levels peaked at 30 min postprandial and were 55.5 ± 7.4 μU/dL in the rice-first group versus 38.2 ± 10.7 μU/dL in the apple-first group. The apple-first group tended to have lower insulin secretion than the rice-first group (though not significant). Similarly, the apple-first group showed a more moderate decrease in insulin levels after 30 min postprandial than the rice-first group.

### 3.2. Changes in the Cumulative iAUC of Blood Glucose and Insulin in Normal Subjects

**Figure 2:**
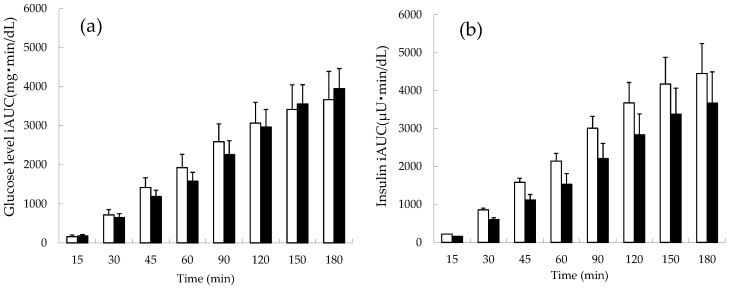
Cumulative incremental area under the curve (iAUC) of blood glucose and insulin in healthy normal subjects. (**a**) Blood glucose. (**b**) Insulin. □: rice-first group, ■: apple-first group (*n* = 10). No significant difference was noted between the rice-first and apple-first groups.

The cumulative iAUC of blood glucose and insulin in normal subjects is shown in Figure 2. The apple-first group showed a tendency for their levels to decrease until 120 min after the meal compared to the rice-first group, after which both groups showed a similar trend, but this was not statistically significant (*p* = 0.63) (Figure 2a). Throughout the study, a trend toward a lower cumulative insulin iAUC was observed in the apple-first versus rice-first group (Figure 2b). In particular, at 60 and 90 min postprandial, the apple-first group tended to suppress insulin secretion (though not statistically significant, *p* = 0.11).

### 3.3. Changes in Blood Glucose Levels and Cumulative iAUC of Blood Glucose in Patients with Impaired Glucose Tolerance

**Figure 3:**
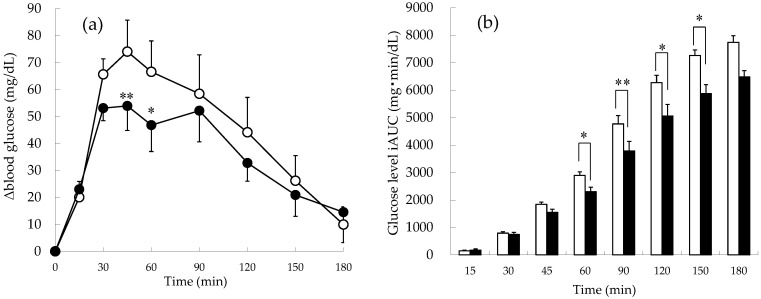
Postprandial blood glucose trends and cumulative incremental area under the curve (iAUC) in patients with impaired glucose tolerance. (**a**) Blood glucose level. (**b**) Cumulative iAUC value of blood glucose. ○: rice-first group, ●: apple-first group, □: rice-first group, ■: apple-first group. * *p* < 0.05; ** *p* < 0.01 (*n* = 9).

Changes in postprandial blood glucose levels and the cumulative iAUC of blood glucose in subjects with impaired glucose tolerance are shown in Figure 3. The blood glucose levels of the apple-first group were lower than those of the rice-first group during the study period (Figure 3a). Interestingly, at 45 and 60 min, when the increase in blood glucose was significant, the apple-first group showed a significant decrease compared to the rice-first group (45 min: 74.1 ± 11.7 mg/dL vs. 53.9 ± 9.1 mg/dL (*p* = 0.001); 60 min: 66.6 ± 11.5 mg/dL vs. 46.8 ± 9.8 mg/dL (*p* = 0.036)). On the other hand, the cumulative iAUC of blood glucose in the apple-first group was lower than in the rice-first group (Figure 3b). In particular, from 60 to 150 min after the meal, the apple-first group showed significantly lower blood glucose than the rice-first group, not that it decreased (*p* = 0.02, 0.0096, 0.03, and 0.045).

### 3.4. Dynamic Parameters in Normal and Impaired Glucose Tolerance Subjects

The dynamic parameters of blood glucose and insulin levels in normal subjects are shown in Table 2. In normal subjects, ΔCmax tended to be lower in the apple-first group compared to the rice-first group (*p* = 0.13). There was no significant intergroup difference in iAUC (*p* = 0.63). In normal subjects, there was a trend toward lower peak insulin levels in the apple-first versus rice-first group for ΔCmax (*p* = 0.10). The iAUC appeared to decrease visually but was not statistically significant (*p* = 0.10), and so we speculated that there was probably less insulin secretion. Tmax was delayed in the apple-first group versus the rice-first group (*p* = 0.27), similar to the results for Tmax in normal subjects.

Table 3 shows the glucose kinetic parameters in subjects with impaired glucose tolerance. ΔCmax was significantly lower in the apple-first group than in the rice-first group (*p* = 0.02). The iAUC of the apple-first group was lower than that of the rice-first group, and blood glucose secretion tended to be suppressed in the former (*p* = 0.09). The Tmax did not differ significantly between the two groups, nor did the timing of intake (*p* = 0.48).

### 3.5. Determination of Carbohydrate (Glucose, Fructose, and Sucrose) Contents

The glucose, fructose, and sucrose contents of the Fuji apples used in this study were as follows: glucose, 2.9 g/100 g; fructose, 7 g/100 g; and sucrose, 2.1 g/100 g. The LOD and LOQ were: glucose, 0.6 and 1.9 ng/mL; fructose, 1.7 and 5.2 ng/mL; and sucrose, 2.5 and 7.7 ng/mL.

### 3.6. Determination of Polyphenol Contents

The total amount of polyphenol in the apples used in this project was 349.0 ± 22.6 mg/100 g (*n* = 3).

## 4. Discussion

In the present study, we observed that apple consumption before meals (the apple-first group) did not exhibit any significant difference in blood glucose and insulin levels when compared with the rice-first group in normal subjects. However, consuming apples first showed a slower increase in the post-prandial blood glucose for the subjects with impaired glucose tolerance. Krishnamachar et al. reported that people who frequently ate apples tended to have less of a postprandial increase in blood glucose and longer-lasting satiety [23]. Interestingly, those who regularly exercise and ate apples as part of their daily routine had the lowest postprandial increase in blood glucose levels. Apples contain soluble dietary fiber (pectin) [26], fructose [27], and alpha-glucosidase inhibitors [28]. Soluble dietary fiber is not easily digested and swells in the stomach, increasing the viscosity of the stomach contents and prolonging the residence time in the stomach, thereby delaying the sugar absorption [29].

Carbohydrates are ingested and absorbed from the intestinal tract and transported through the portal vein, first to the liver and then to the rest of the body. Carbohydrates in the body are supplied to the various tissues as a source of energy and then stored as glycogen in the liver and muscles. Fructose, a monosaccharide, is incorporated into the metabolism of glucose (glycolysis) but follows a different pathway in the muscles and liver. Fructose is converted to triglycerides in the liver, which can increase very low-density lipoproteins, leading to hyperlipidemia. However, most of the metabolism of fructose reportedly occurs in the cells of the small intestine, where it is first phosphorylated by ketohexokinase and then passed downstream [30]. In the present study, the Tmax value of blood glucose was prolonged in the group that consumed apples first, regardless of whether they had impaired glucose tolerance. Other possible effects on blood glucose levels may be due to pectin, a type of dietary fibre found in apples [26]. In addition, the prolongation of Tmax was more pronounced in those with impaired glucose tolerance than in those with normal glucose tolerance, possibly due to a slower cellular uptake of glucose.

The blood glucose Cmax levels in both subject groups were reduced by the pre-meal consumption of apples (the apple-first group). These results may be due to the inhibition of glucose and, hence, the carbohydrate digestion and absorption processes by alpha-glucosidase inhibitors and polyphenols. Apple peel contains alpha-glucosidase inhibition, and apple flesh contains amylase inhibitors, which may be a benefit for managing hyperglycemia while reducing side effects [30,31]. The polyphenols and other compounds found in apples are known as phytochemicals [32]. Phytochemicals are thought to scavenge reactive oxygen species, which promote aging and can lead to T2D and cancer. In this study, the flavonoid content was determined to be 55.6 ± 3.6 mg/100 g (*n* = 3); thus, we believe that apples will provide valuable information in the management of diabetes. In particular, when nutritionists and pharmacists give advice and nutritional guidance to healthy people on their daily diet with an awareness of unwellness, they may be able to provide recommendations on how to consume apples.

The carbohydrate analysis in this study confirmed that apples contain 7.0 g/100 g of fructose. Fructose, the major carbohydrate found in apples [27], is reportedly absorbed more slowly in the intestine and more rapidly in the liver [32] due to differences in absorption and the metabolic pathway compared to glucose [33]. This will result in a slower increase in blood glucose levels after meals. Fructose in the liver is mainly metabolized to glucose and triglycerides, but most of it is quickly stored as glycogen [31], resulting in fructose causing only a slight increase in blood glucose levels after a meal. Therefore, the consumption of apples before meals does not increase the postprandial blood glucose even if glucose intolerance is induced; rather, it inhibits the absorption of sugar and moderates the secretion of insulin. In this study, however, we did not measure the insulin levels of people with impaired glucose tolerance, as the amount of plasma collected was insufficient.

Due to only having 10 subjects per group, availability of sufficient evidence may be limited, but we believe that it will help provide valuable information on blood glucose management. People with impaired glucose tolerance generally have reduced insulin secretion and cellular uptake [34], which may be the result of the multifaceted effects of fructose, dietary fiber gastric emptying, apple composition during gastric digestion [35,36], and α-glucosidase inhibitors on the insulin-independent increase in blood glucose levels.

This study used 150 g of apples. Normally, 100 g of apple (about 1/3 of a medium apple) contains about 50 kcal and about 10 g of carbohydrate. These calories and carbohydrates are equivalent to about 1/3 of a rice bowl, the same amount of rice. From this point of view, apples are a low GI food, and these preliminary findings support further investigation of the consumption of apples to support glycemic control. Finally, the study has some limitations. Having two individuals with impaired glucose tolerance drop out of the study made the researchers realize the difficulties of the study’s design. The condition of patients with impaired glucose tolerance changes on a day-to-day basis. We, as medical researchers, had the opportunity to ponder what approaches to health maintenance and promotion can contribute to healthcare in the future. The results of this study are also expected to help physicians, pharmacists, and other healthcare professionals in providing health support to community residents and those with impaired glucose tolerance.

## 5. Conclusions

This study showed that apple consumption before meals could improve postprandial hyperglycemia in normal subjects and those with impaired glucose tolerance. This information is simple and easily disseminated and may be useful for the development of dietary guidance for type II diabetic patients in the future.

## Figures and Tables

**Table 1:** Nutritional components and test food amounts.

	Total (g)	Protein (g)	Fat (g)	CHO A Vial (g)	Energy (kcal)
Rice	147.5	3.1	0.6	50.0	223.2
Apple	150.0	0.3	0.2	21.9	80.0
Total	297.5	3.4	0.8	71.9	303.2

https://rnavi.ndl.go.jp/mokuji_html/000003076054.html (accessed on 23 May 2022).

**Table 2:** Dynamic parameters of blood glucose and insulin levels in the normal subjects.

Glucose Level	ΔCmax (mg/dL)	Tmax (min)	iAUC (mg·min/dL)
Rice-first group	54.0 ± 5.0	33.0 ± 2.0	3666 ± 458
Apple-first group	46.2 ± 5.9	54.0 ± 12.7	3952 ± 652
**Insulin Level**	**ΔCmax** **(µU/dL)**	**Tmax** **(min)**	**iAUC** **(µU·min/dL)**
Rice-first group	61.9 ± 7.2	31.3 ± 4.2	4452 ± 878
Apple-first group	49.8 ± 8.5	45.0 ± 9.2	3669 ± 916

Mean ± SEM, *n* = 10. No significant difference was noted between groups.

**Table 3:** Dynamic parameters of blood glucose levels in the impaired glucose tolerance subjects.

Glucose Level	ΔCmax (mg/dL)	Tmax (min)	iAUC (mg·min/dL)
Rice-first group	90.0 ± 10.0	48.3 ± 6.5	7739 ± 1474
Apple-first group	75.2 ± 7.2 *	55.0 ± 9.0	6481 ± 1105

iAUC, incremental area under the curve. Mean ± SEM, *n* = 10. * *p* < 0.05: rice-first versus apple-first group.

## Data Availability

Data are contained within the article.
